# Contribution of Milk Beverages to Nutrient Adequacy of Young Children and Preschool Children in the Philippines

**DOI:** 10.3390/nu12020392

**Published:** 2020-02-01

**Authors:** Tsz-Ning Mak, Imelda Angeles-Agdeppa, Marie Tassy, Mario V. Capanzana, Elizabeth A. Offord

**Affiliations:** 1 Nestlé Research, Route du Jorat 57, 1000 Lausanne, Switzerland; Marie.Tassy@rd.nestle.com (M.T.); elizabeth.offord-cavin@rdls.nestle.com (E.A.O.); 2 Food and Nutrition Research Institute-Department of Science and Technology, Taguig City 1631, Philippines; iangelesagdeppa@yahoo.com.ph (I.A.-A.); mar_v_c@yahoo.com (M.V.C.)

**Keywords:** milk, dairy, nutrient adequacy, Philippines, young children, preschool children

## Abstract

Malnutrition is a major public health concern in the Philippines. Milk and dairy products are important sources of energy, protein, and micronutrients for normal growth and development in children. This study aims to assess the contribution of different types of milk to nutrient intakes and nutrient adequacy among young and preschool children in the Philippines. Filipino children aged one to four years (*n* = 2992) were analysed while using dietary intake data from the 8th National Nutrition Survey 2013. Children were stratified by age (one to two years and three to four years) and by milk beverage consumption type: young children milk (YCM) and preschool children milk (PCM), other milks (mostly powdered milk with different degrees of fortification of micronutrients), and non-dairy consumers (no milks or dairy products). The mean nutrient intakes and the odds of meeting nutrient adequacy by consumer groups were compared, percentage of children with inadequate intakes were calculated. Half (51%) of Filipino children (all ages) did not consume any dairy on a given day, 15% consumed YCM or PCM, and 34% consumed other milks. Among children one to two years, those who consumed YCM had higher mean intakes of iron, magnesium, potassium, zinc, B vitamins, folate, and vitamins C, D, and E (all *p* < 0.001) when compared to other milk consumers. Non-dairy consumers had mean intakes of energy, total fat, fibre, calcium, phosphorus, iron, potassium, zinc, folate, and vitamins D and E that were far below the recommendations. Children who consumed YCM or PCM had the highest odds in meeting adequacy of iron, zinc, thiamin, vitamin B6, folate, and vitamins C, D, and E as compared to other milks or non-dairy consumers, after adjusting for covariates. This study supports the hypothesis that dairy consumers had higher intakes of micronutrients and higher nutrient adequacy than children who consumed no milk or dairy products. Secondly, YCM or PCM have demonstrated to be good dairy options to achieve nutrient adequacy in Filipino children.

## 1. Introduction

A double burden of malnutrition exists in the Philippines. The prevalence of undernutrition in children is high, where one in three children under five years are stunted and 7% are wasted [1]. Micronutrient deficiency continues to be a key public health concern; vitamin A deficiency affects one in five children, while 14% and 18% of children < 5 years are anemic and zinc deficient, respectively [2].

A previous study found that children in the Philippines have poor diet diversity, and the prevalence of inadequate intakes of micronutrients is high [3]. Seventy-eight percent, 75% and 90% of Filipino children aged one, two, and three to four years, respectively, had inadequate intakes in iron; 62%, 66%, and 84% were inadequate in calcium; 52%, 46%, and 47% were inadequate in zinc, 60%, 41%, and 43% were inadequate in vitamin A, respectively. More than 40% of children aged one year were inadequate in B vitamins (thiamine, riboflavin, niacin, B6, folate, and B12), and in children three to four years, 72% were inadequate in folate and 60% in vitamin C [3]. The diet of children in the Philippines typically composed of refined rice and energy-dense foods such as cookies and sugar sweetened beverages, low in fruit, vegetables, and protein-rich foods [3]. The consumption of milk and dairy products is generally low among children in the Philippines with a decreasing trend with age [3]. At age one year, 37% of Filipino children were having breastmilk, 35% consuming cow’s milk and 23% consuming toddler milk; at two years, only 11% were receiving breastmilk, 38% of children were consuming cow’s milk, and 15% consuming toddler milk; and, at age three to four years, only 32% of children would consume milk on a given day [3].

Milk and dairy are part of an important food group for preschool children, as it greatly contributes to total energy, protein, and calcium in relation to children’s daily requirements for normal growth and development [4]. In the Philippines, powdered milk is the most common type of milk available. It is a milk usually intended for the whole family, and is typically fortified with calcium and vitamin A. Young children milk (YCM) and preschool children milk (PCM), are milks tailored for children’s nutritional needs at different age stages, fortified with vitamins and minerals, some with DHA and probiotics. A number of observational studies and randomized controlled trials (RCT) in Europe and Australia and New Zealand have demonstrated that, when compared to cow’s milk, YCM improved nutrient intakes among children above 12 months, particularly in iron and vitamin D [5,6,7,8,9]. Several studies have shown that children who consumed YCM had better iron and vitamin D status than non-consumers [7,10,11], and it had a lower percentage of body fat than children who consumed cow’s milk [12]. This is likely due to the lower protein of YCM than cow’s milk, as evidence supports the early protein hypothesis that high protein intake in early years might promote weight gain and higher risk of obesity in later life [13,14].

Beyond developed countries, there is a lack of studies on the impact of YCM or PCM in children. A RCT on Indian children found that those who consumed fortified milk had improved weight and height gain and iron status than those who consumed unfortified milk [15]. In the Philippines, only one study can be found to measure the impact of fortified milk among young children. The clinical study showed that preschool children (three to five years) from a small town in Philippines, who consumed two servings of fortified milk a day for 12 weeks significantly increased the intakes of energy, protein, iron, vitamin A, calcium, thiamin, riboflavin, niacin, and vitamin C when compared to baseline [16]. Therefore, more evidence is needed to examine the role of milks in the diet of children across the Philippines, where nutrient inadequacy is highly prevalent.

The aim of the current study was therefore to assess the contribution of different milk types to nutrient intakes and nutrient adequacy among young children and preschool children in the Philippines. We hypothesized that children who consumed milk (YCM/ PCM/ other milks) would have higher intakes of micronutrients and higher nutrient adequacy than children who consumed no milk or dairy products. Furthermore, we also hypothesized that YCM/ PCM consumers may have higher nutrient adequacy than other milk consumers, given the high level of fortification of micronutrients in YCM/PCM.

## 2. Materials and Methods

### 2.1. Study Population

Dietary intake data of children aged one to two years (*n* = 1461) and three to four years (*n* = 1531) from the 2013 National Nutrition Survey (NNS) were analysed. The details of the NNS have been reported previously [17]. The 2013 NNS was a cross-sectional nationally representative health and nutrition survey of the Filipino population. Filipino households (*n* = 35,825) were sampled with a response rate of 91% [3]. The Ethics Committee of Food Nutrition Research Institute (FNRI) approved the survey protocol and data collection instruments. All of the surveyed households provided informed consent prior to participation with a registry number FIERC—2013—001 [17].

### 2.2. Data Collection

Trained dietitians conducted face-to-face 24-h dietary recalls with a parent or caregiver of each child during household visits, wherein the dietitian recorded all food and beverages that the child consumed the previous day. A first 24-h recall was performed on all children and a second 24-h recall was repeated in 50% of randomly selected households, typically two days after the first recall. The amount of each food item or beverage was estimated while using common household measures, such as cups, tablespoons, by size, or number of pieces. The information was then converted to grams while using a portion-to-weight list for common foods compiled by FNRI or through weighing of the food samples. For powdered milk, a conversion factor of 6.7 was multiplied to estimate the gram weight of fluid milk as consumed, based on the preparation instruction of approximately 36 g milk powder reconstituted to approximately 240 g of fluid milk (in line with recommendation from the Philippines Nutritional Guide Pyramid—four tablespoons of milk powder to one glass of water) [18]. All of the reported foods and drinks were reviewed, to ensure that all of the codes and quantities were correctly entered. The food and beverages consumed were converted to energy and nutrient intakes while using the Philippines Food Composition Tables (PFCT) [19]. The energy intake distribution of the full sample (*n* = 3016) was assessed and the outliers were defined as energy intakes three standard deviations above and below (±3SD) the mean per age group (one to two year and three to four years) and were removed from analyses. A final sample size of 2992 was retained for analysis.

The 888 unique food items reported were categorised into nine major food groups and further subgroups. The food grouping system was adapted from What We Eat in America Food Categories [20] and US Feeding Infants and Toddlers Study (FITS) adjusted for the Philippines local food culture [3].

Demographic characteristics, including dwelling location, wealth quintiles, mother’s education, occupation status, and civil status were collected. Children’s height-for-age and weight-for-age were obtained and the BMI z-scores were calculated to define their nutritional status (wasting, at risk of overweight, and overweight).

### 2.3. Data Processing

The three consumer groups were defined as follow. Children who reported to have consumed “Formula”, which included subcategories “Toddler/Pre-school formula” and “Infant formula” were considered as “YCM or PCM consumers”. We identified only 6% of children at age 1 year who were still consuming infant formula. Children who had reported to consume “Milk” which included subcategories “Milk, powdered”, “Milk, fluid”, “Other Milk”, and “Dairy products” were categorized as “Other milk consumers”. Dairy products (cheese and yogurt) consumption was only represented by 2.7% of the sample and was therefore grouped together with the milk subcategories. Children who had consumed both YCM/PCM and other milks were considered as “YCM/PCM consumers”. All of the children who did not consume any of the above (YCM/PCM, other milk or dairy) were considered “non-dairy consumers”. The nutritional compositions of YCM/PCM, milk (fluid), and milk (powdered) considered in the analyses can be found in Supplementary Materials Table S1.

Twenty-seven nutrients were available in the PFCT. After checking for completeness, 22 nutrients, including total energy (kcal), total carbohydrates (g), fibre (g), protein (g), total fat (g), sodium (mg), calcium (mg), phosphate (mg), iron (mg), magnesium (mg), potassium (mg), selenium (mg), zinc (mg), thiamine (mg), riboflavin (mg), niacin (mg), vitamin B6 (mg), folate (µg), vitamin B12 (mg), vitamin C (mg), vitamin D (µg), and vitamin E (mg) were analysed. Vitamin A, total sugars, saturated fat, monounsaturated fat, and polyunsaturated fat, while available in the PFCT, had high percentage of missing values for the food and beverages of interest, which were deemed too incomplete for the current analysis. The total daily intake of each nutrient based on food consumption was calculated per person per day. An average of nutrient intakes over the two days were considered in the subsequent analyses for children who had a second 24 h recall day.

### 2.4. Statistical Analysis

All analyses were stratified by age groups: one to two years, and three to four years. The relationships between sociodemographic variables (gender, age group, wealth quintiles, dwelling location, mother’s education, civil status, and occupation status, and BMI z-scores) and consumer groups were tested for significance while using chi-squared tests and Kruskal–Wallis tests. The mean intake per nutrient per consumer group was calculated. Non-parametric Kruskal–Wallis tests were used to compare the mean ranks of each nutrient between three consumer groups due to the skewness of the nutrient intake data. Post hoc Dunn’s tests were then performed to compare mean nutrient intakes between YCM/PCM consumers and other milk consumers, and YCM/PCM consumers and non-dairy consumers.

Inadequate intakes were defined while using the estimated average requirements (EAR) cut-off method. Children with intake below the EAR for a given nutrient were considered to be inadequate. For nutrients that did not have an established EAR cut-off, e.g., vitamin D and vitamin E, adequate intake (AI) values were used. The Philippines Nutrient Reference Intakes (PNRI) table was used to establish EAR/AI for protein, calcium, phosphorus, iron, selenium, zinc, thiamin, riboflavin, niacin, vitamins B6, B12, folate, and vitamins C, D, and E. For macronutrients (except for protein where EAR is available), Acceptable Macronutrient Distribution Ranges (AMDR) were used to evaluate carbohydrates and total fat, as a percentage of energy. The proportions of inadequate intakes were classified as %E less than the AMDR lower range. The proportion of children with inadequate intakes were calculated per consumer group.

Logistic regression analysis was used to estimate the odds ratios of children meeting the EAR/AI/AMDR of each nutrient with respect to the consumer group, to test the hypothesis that YCM/PCM consumers (reference group) had higher odds of meeting the EAR/AI/AMDR of nutrients when compared to other milk consumers and/or non-dairy consumers. Adjustment of potential confounding factors—wealth quintiles, dwelling location, mother’s education, and mother’s occupation was included in the final model. R version x64.3.6.1 and R-Studio version 1.2.1335 were used for all of the statistical analysis.

## 3. Results

### 3.1. Descriptive Statistics

Table 1 shows a description of the sample population by consumption group. There was an equal split in gender within the sample. Eleven percent of children one to two years suffered from wasting, while 13% were at risk of overweight or overweight. The majority came from the rural area and in poor to poorest wealth quintiles. Most mothers were not working, were married, and were largely high school graduates. There were no significant differences between consumption groups and gender, BMI z-scores, and mother’s civil status. However, wealth quintiles, mother’s education, and mother’s occupation status were significantly related to if children consumed YCM/PCM, other milk or no dairy (all *p* < 0.001).

Of the 1461 children aged one to two years, 43% had no dairy (other milk, YCM or other dairy products), 35% were other milk consumers, and 22% YCM consumers. The mean consumption of YCM before reconstitution was 101 g/d (±SD 69 g) and in fluid weight was 641 g/d (±SD 432 g); mean consumption of other milk before reconstitution was 61 g/d (±SD 58 g) and in fluid weight was 354 g/d (±SD 330 g).

Among children aged three to four years (*n* = 1531), the majority (58%) did not consume any dairy, 34% consumed other milks, and 8% of children consumed PCM. Mean consumption of PCM before reconstitution was 85 g/d (±SD 73 g) and, in fluid weight, was 454 g/d (±SD 355 g); mean consumption of other milks before reconstitution was 46 g/d (±SD 53 g) and in fluid weight 224 g/d (±SD 233 g) for this age group.

### 3.2. Comparison of Mean Nutrient Intakes by Consumer Group

#### 3.2.1. YCM/PCM Consumers vs. Other Milk Consumers

Table 2 shows the comparison of mean nutrient intakes by consumer group. YCM/PCM consumers and other milk consumers had similar macronutrient intakes, except for protein (one to two years only), where YCM consumers had lower intake. YCM/PCM consumers had higher total fat (three to four years only) and higher micronutrient intakes than other milk consumers, except for sodium, phosphorus, riboflavin, and selenium (one to two years only). No significant differences were found for calcium intakes in children aged one to two years. No significant differences were found for sodium, magnesium, selenium, and B12 in the older children.

YCM/PCM and other milk consumers both had mean intakes of energy, fibre, and potassium lower than the recommended intake levels. In general, YCM/PCM consumers had mean intakes of micronutrients closer to the recommendations compare to other milk consumers. In children aged one to two years, other milk consumers had mean intakes of iron, magnesium, folate, and vitamins D and E far below the recommendations.

#### 3.2.2. YCM/PCM Consumers vs. Non-Dairy Consumers

Non-dairy consumers had significantly lower intakes than YCM consumers in most macro- and micronutrients, except for fibre in children aged one to two years (*p* < 0.05). No significant differences were seen for carbohydrates, fibre, and magnesium and selenium between the PCM and non-dairy consumer groups in children aged three to four years. Non-dairy consumers had mean nutrient intakes far below the recommendation. The only nutrients where non-dairy consumers were in line or above the recommended levels were protein, sodium, selenium, and niacin (all ages); magnesium, B6 and B12 (three to four years only).

### 3.3. Percentage of Children with Inadequate Intakes

Table 3 shows the proportion of children with nutrient intakes below the EAR or AI across the three consumer groups. Among non-dairy consumers aged one to two years, except for carbohydrates and selenium, more than 50% of children had intakes below the EAR/AI. In particular, almost all children in this group were inadequate in calcium, vitamin D, and iron (99%, 97% and 91%, respectively). The highest level of inadequate intakes among other milk consumers (one to two years) were vitamin D (98%), vitamin E (97%), iron (92%), folate (82%), and vitamin B6 (73%). YCM consumers (1 to 2 years) had the lowest percentage of inadequate intakes when compared to the other groups. The highest levels of inadequacy in this group were vitamin E (68%), phosphorus (55%), vitamin D (52%), and folate (49%).

Among preschool children, the proportions with inadequate intakes were slightly lower than the younger children, except for calcium in PCM (67%) and other milk consumers (65%), and vitamin D (72%) in PCM consumers. Non-dairy consumers still had the highest proportion of children with inadequate intakes across most nutrients.

### 3.4. Odds of Nutrient Adequacy Per Consumer Group

Table 4 illustrates the odds ratios of other milk consumers and non-dairy consumers meeting nutrient adequacy when compared to YCM/PCM consumers (reference group), after adjusting for potential confounding factors (wealth quintiles, dwelling location, mother’ education level, mother’s occupation). Among children aged one to two years, other milk consumers were significantly less likely to meet the AMDR for carbohydrates, as well as the EARs for iron, zinc, thiamin, niacin, vitamin B6, folate, vitamins C, D, and E (all *p* < 0.001), and vitamin B12 (*p* = 0.002), than YCM consumers (reference group). Other milk consumers were more likely to meet the EAR of protein (*p* = 0.003), phosphorus, selenium, and riboflavin (*p* ≤ 0.001) than YCM consumers. Non-dairy consumers had significantly lower odds of meeting the adequacy of all nutrients when compared to YCM consumers, except for carbohydrates and selenium (both non-significant).

In preschool children, other milk consumers were less likely to reach nutrient adequacy in iron, folate, vitamins C, D, E (all *p* < 0.001), zinc, thiamin, and vitamin B6 (all *p* < 0.05), but were more likely to meet adequacy in phosphorus and riboflavin when compared to PCM consumers. Non-dairy consumers had lower likelihood of meeting adequacy in total fat, calcium, iron, zinc, folate, vitamins C, D, E (all *p* < 0.001), and thiamin (*p* = 0.005) and vitamins B6 (*p* = 0.024), but more likely of being within AMDR of carbohydrates (*p* = 0.016) than PCM consumers.

The results also highlight the sociodemographic factors that were significantly related to increased or reduced odds of meeting nutrient adequacy. In children one to two years, living in urban areas increased the likelihoods of meeting protein, phosphorus, zinc, thiamin, niacin, and vitamin B6 adequacies; being in the richest wealth quintile were associated with adequacy in total fat, calcium, zinc, thiamin, and vitamin B6 (all *p* < 0.05). On the other hand, being in poor or poorest wealth quintiles, and/or mothers with education at or below high school levels reduced the odds of meeting adequacy in most nutrients, including protein, total fat, calcium, selenium, zinc, B vitamins, and vitamin E (all *p* < 0.05). Similar trends were also observed in children aged three to four years. The only nutrient that had an opposite trend than the rest of the nutrients was carbohydrate. In children aged one to two years, being in the richest wealth quintile lowered the odds of meeting the AMDR for carbohydrates, and similarly among three to four years, those who were the richest, living in urban dwelling, and with mother currently working reduced the likelihood of meeting AMDR for carbohydrates, while being in the poor or poorest wealth quintile increased the odds.

None of the sociodemographic factors were associated with iron, vitamins C and D adequacies in children aged one to two years, and vitamin B6, folate, vitamins D and E adequacies in children three to four years.

## 4. Discussion

The current study assessed the contribution of different milks to daily nutrient intakes among young and preschool children in the Philippines, by comparing the mean nutrient intakes and percentage of children with inadequate intakes between three consumer groups: YCM/PCM consumers, other milk consumers, and non-dairy consumers. Our analysis has shown that children in the Philippines who consumed dairy products, including YCM/PCM and other milks, had higher intake of most nutrients and lower nutrient inadequacy than non-dairy consumers. Non-dairy consumers, in particular, had intakes of energy, total fat, calcium, phosphorus, iron, potassium, folate, vitamin D, and vitamin E far below recommendations. On the other hand, YCM/PCM consumers had mean intakes of micronutrients that were closer to the recommendations when compared to other consumer groups. While other milk consumers had similar macronutrient intakes to YCM/PCM consumers, a higher percentage of other milk consumers had inadequate micronutrient intakes as compared to YCM/PCM consumers. These findings support the fact that dairy is an important food group that significantly contributes to the intakes of children in these two age groups. Moreover, the choice of dairy products (e.g., YCM/PCM vs. other milks) contributes differently to total daily nutrient intakes, and that YCM/PCM is a good option in terms of reducing micronutrient inadequacy.

### 4.1. Nutrient Inadequacy in Filipino Children

The alarming prevalence of inadequate intakes, particularly for energy and micronutrients, among young children in the Philippines deserves further attention. Overall, the energy intakes of the three consumer groups were below the age and gender specific Recommended Energy Intakes in the Philippines. Moreover, 77% of children (one to four years) were inadequate in calcium, 81% inadequate in iron, 74% in folate, 60% in zinc, 62% in vitamin C, and 90% in vitamin D. Previous studies have found that the diets of young Filipino children lacked diversity, which contributed to high levels of nutrient inadequacy [21,22]. While improving diet diversity (increasing number of food groups consumed) could incrementally increase micronutrient adequacy, a more recent study found that, even with high diet diversity, Filipino school children had difficulty in achieving adequacy in calcium, folate, iron, vitamin A, and vitamin C. This is likely due to the low quantities of food consumed, or that the current food supply in the Philippines might not contain enough of these nutrients to fulfil the needs of children [23]. Indeed, our logistic regression analysis provided interesting insights that those living in urban areas and wealthier socio-economic quintiles were more likely to achieve total fat and micronutrient adequacies, while those from poorer socio-economic backgrounds and having mothers with lower education levels had reduced odds of meeting micronutrient adequacies, except for carbohydrates. It is possible that those from higher socio-economic quintiles ate better diets and that living in urban areas meant better accessibility and availability of more nutritious foods (e.g., meat) and fortified beverages; and, those from lower socio-economic quintiles had a predominately carbohydrate-rich (e.g., rice) diet that lack variety. Our findings support the literature on key strategies to reduce stunting in Southeast Asia [24] and in Filipino children [23]. There is an urgent need for better access to a variety of nutrient-rich foods, particularly in the lower socio-economic groups, increase availability of fortified foods that are tailored for young children, in tandem with increased parental education on dietary intake, to close nutrient gaps among young children. 

### 4.2. Large Proportion of Filipino Young Children Did Not Consume Dairy on a Given Day

The Daily Nutritional Guide Pyramid for Filipino Children aged one to six years recommends consuming one glass of milk & milk products per day, which includes one glass of whole milk, or 1/2 cup evaporated milk diluted with 1/2 glass water, or four tablespoons of powdered whole milk diluted with one glass of water [18]. However, the current study found half of Filipino children (43% aged one to two years and 58% aged three to four years) were not consuming any milk or milk products on a given day. Non-dairy consumers had mean energy intakes approximately 40% lower than the Recommended Energy Intakes (REI) (one to two years: mean intake of 543 kcal/d vs. REI of 1000 kcal/d for boys and 920 kcal/d for girls; three to four years: mean intake of 828 kcal/d vs. REI of 1350 kcal/d (boys) and 1260 kcal/d (girls)), suggesting these young children were at serious risk of undernutrition.

Indeed, the consumption of milk and animal-sourced foods is known to be limited among low-income countries. It is estimated that animal-source foods, such as milk, provide between 5% to 15% of total daily energy in Asian countries, when compared to over 20% daily energy in western countries, such as the U.S. and Europe [4]. A study on South East Asian countries, including Thailand, Malaysia, Vietnam, and Indonesia also observed sub-optimal milk and dairy intake in children aged one to 12 years. Only around half of Indonesian (52%) and Vietnamese (47%) children consumed dairy products on a daily basis. The study found that children who consumed less than one portion of dairy per day had significantly lower nutrient intakes and higher prevalence of underweight and stunting than children who consumed ≥1 portion of dairy per day [25].

Our analysis also suggests that a high percentage of non-dairy consumers came from poorer wealth quintiles, from rural areas, and with mothers who had lower education levels; while, the opposite sociodemographic characteristics were observed for those who consumed YCM/PCM. Financial constraints, limited product availability, and lack of parental awareness of the importance of nutrition and dairy food could be the reasons for the high proportion of children not consuming YCM/PCM or other milks.

### 4.3. The Role of YCM/PCM in the Diet of Filipino Children

A previous study has highlighted that both cow’s milk and YCM/PCM were two of the top five contributors of energy, carbohydrate, protein, fat, thiamine, riboflavin, vitamins A, C, calcium, iron and zinc for Filipino children aged 12 to 35.9 months [3]. Particularly, YCM was a top contributor of iron and zinc in children aged 12–23.9 months. Other studies in Europe and Oceania found that YCM/PCM consumers had higher intakes of nutrients, such as iron, vitamins C, and D than cow’s milk consumers [5,6,7,8]. Our study adds to the evidence that YCM/PCM and other milk consumers had nutrient intakes that were more in line with recommendations than non-dairy consumers, and that the dairy food group can contribute hugely to the daily intake of nutrients in young children. Furthermore, the fact that lower percentages of YCM/PCM consumers had inadequate intakes, and that they had higher odds of achieving adequacy in iron, zinc, thiamine, niacin, folate, vitamins B6, B12, C, D when compared to other milk consumers, suggests that YCM/PCM might be a good choice of dairy product for Filipino children.

In contrast to the expert opinions and recommendations on toddler milk and YCM from Europe and the U.S. [13,26,27,28], the routine use of YCM is not deemed a necessity, as the missing nutrients in the diet of young children can also be provided by other dietary sources in these countries, the findings of the current study did support the role of YCM in the diet of young children in the Philippines. Furthermore, our study also supported that the use of YCM/PCM can increase the intake of iron and vitamin D and decrease the intake of protein when compared to other milk, in accordance to the European Food Safety Authority (EFSA) scientific opinion [26], conclusion from the European Society for Paediatric Gastroenterology Hepatology and Nutrition (ESPGHAN) committee [13], and findings from previous studies [5,6,7,8,9,10,11].

However, one caution to highlight is that, while our data supports that, overall, YCM/PCM consumers have lower nutrient inadequacies than other consumer groups, not all YCM/PCMs on the market have favorable nutritional compositions for children. Table S1 highlights the minimum, maximum, and mean nutrient compositions across 18 brands of YCM/PCMs. Parents and health care professionals should consider the nutritional profiles of YCM/PCMs when choosing YCM/PCM for children, as, evidently, YCM/PCMs have highly variable macro- and micronutrient content, and not all are fortified with essential micronutrients to the same extent.

### 4.4. Strengths and Limitations

The large sample of children (*n* = 2992) from a nationally representative survey of the Philippines covering the young toddlers one to four years is a strength of this study. The nutrient intakes of this age group have not been extensively studied in this country or in the Southeast Asia region, and this study has added to the existing literature that dairy is a key food group to the diet of young Filipino children beyond infancy. This study, to our knowledge, is the first to identify the high percentage of young children who are not consuming any dairy in the Philippines. This highlights the opportunity for the public health authority in the Philippines to improve dairy consumption in children.

One limitation of the current study is the method for estimating total quantity of powdered milk and YCM/PCM consumed. Powdered milk and YCM/PCM consumption were recorded as gram weight in powder form in the survey and a conversion factor of 6.7 was applied to estimate fluid weight (g), as consumed. While we had checked several milk brands’ on-pack preparation instructions, and the conversion the factor used was deemed appropriate, it is possible that the total quantity of other milk and YCM/PCM as consumed was overestimated. Indeed, the estimated total YCM consumed by children aged one to two years (641 g/d) was higher than anticipated. A report of an expert panel recommended two to three servings, with a total of 400–600 mL of YCM/PCM per day are appropriate for children age one to six years [29]. The nutrient composition of the milk or YCM/PCM did not change and would not affect the calculation of mean nutrient intakes or percentage of children with inadequate intakes, despite the conversion to weight as consumed.

Another limitation is that the high proportion of missing data for some of the nutrient variables (vitamin A, saturated fat, monounsaturated, and polyunsaturated fats) meant that they were excluded for analyses. Given that vitamin A inadequacy is known to be high in the Philippines from the literature and that the fatty acids also are of public health importance, it was unfortunate that they were not investigated in the current analysis.

## 5. Conclusions

This study highlights the high prevalence of inadequate intakes, particularly for energy and micronutrients, among young children in the Philippines, regardless of milk consumption type. Secondly, the current study also provides novel insights on the importance of dairy food group including YCM/PCM and other milk to energy and macronutrient intakes, and meeting micronutrient adequacy in the diet of Filipino children. YCM/PCM have demonstrated to be a more superior option than other milks to achieve adequacy in key nutrients, such as iron, zinc, vitamins C, D, E, and some B vitamins in this population. Furthermore, expert opinions and recommendations on the role of YCM/PCM from Europe or the U.S may not apply to countries, such as the Philippines, where nutrient inadequacies and deficiencies are much more common. Finally, strategies targeting specific socio-demographic segments (e.g., by wealth status, dwelling location, and mother’s education) should be adopted to increase the intake of YCM/PCM or other fortified nutrient-dense foods and beverages among young children in the Philippines to improve nutrient intakes and reduce nutrient inadequacy.

## Figures and Tables

**Table 1:** Sample characteristics by consumer group (toddlers and young children aged one to four years).

		YCM/PCM Consumers		Other milk Consumers		Non-Dairy Consumers		Total	*p*-Value
		n	%	n	%	n	%	n	
Gender	Boys	235	16	527	35	745	49	1507	
	Girls	210	14	502	34	773	52	1485	0.362
Age group	1 to 2 years	328	22	506	35	627	43	1461	
	3 to 4 years	117	8	523	34	891	58	1531	<0.001
Wealth quintiles	Poorest	29	3	187	22	648	75	864	
	Poor	37	6	222	36	357	58	616	
	Middle	75	13	233	41	266	46	574	
	Rich	116	24	230	47	142	29	488	
	Richest	175	48	124	34	68	19	367	<0.001
Dwelling location	Rural	169	10	519	31	991	59	1679	
	Urban	276	21	510	39	527	40	1313	<0.001
Mother’s Education	No Grades Completed	1	3	2	6	31	91	34	
	Elementary Level	13	2	137	26	375	71	525	
	High School Level	66	7	324	34	554	59	944	
	Vocational Level	20	23	31	35	37	42	88	
	College Level	134	36	124	34	111	30	369	
	Others	0	0	0	0	5	100	5	<0.001
Mother’s Civil Status	Single	9	14	22	34	34	52	65	
	Married	172	12	434	31	792	57	1398	
	Live-in	43	10	128	31	243	59	414	
	Separated/Annulled/Divorced	1	5	8	40	11	55	20	
	Widow/Widower	2	13	6	40	7	47	15	
	Unknown	0	0	0	0	1	100	1	0.917
Mother’s occupation status	With Job/Business	99	20	165	33	238	47	502	
	Housekeeper/No Occupation/Pensioner/Student	128	9	432	31	850	60	1410	<0.001
BMI z-scores	<−2 (Wasting)	41	16	93	36	121	47	255	
	−2 to −1	54	9	187	33	334	58	575	
	−1 to 0	119	11	371	34	605	55	1095	
	0 to 1	125	17	257	35	342	47	724	
	1 to 2 (At risk of overweight)	66	29	79	34	85	37	230	
	>2 (Overweight)	40	35	42	37	31	27	113	0.495

**Table 2:** Mean and distribution of nutrient intakes by consumer group for children aged one to two years, three to four years.

1 to 2 Years											3 to 4 Years									
	Reference Intake	Mean and Distribution of Intakes									Reference Intake	Mean and Distribution of Intakes								
	M/F		Mean	SD	a	10th	25th	50th	75th	90th	M/F		Mean	SD	a	10th	25th	50th	75th	90th
Energy (kcal)	1000/920 ^1^										1350/1260									
		All	690.2	383.0		253.9	404.7	629.6	901.8	1236.5		All	915.1	395.0		455.6	616.1	860.5	1151.3	1459.5
		YCM	798.4	377.4		343.3	492.1	774.8	1047.8	1347.2		PCM	1058.6	408.5		502.6	817.5	1011.8	1351.4	1538.4
		Other milk	802.2	378.5	N.S.	380.3	532.8	741.1	1004.1	1338.4		Other milk	1031.2	392.1	N.S.	574.0	727.8	974.8	1265.1	1607.6
		Non-dairy	543.3	338.1	*	175.0	290.4	474.2	727.9	989.8		Non-dairy	828.1	371.6	*	410.7	552.6	772.3	1036.0	1318.2
Protein (g)	15/14										18/17 ^1^									
		All	22.3	14.6		6.6	11.6	19.7	30.0	40.6		All	28.8	14.8		12.6	18.3	26.2	36.6	47.1
		YCM	24.6	13.3		9.6	14.9	22.9	33.8	42.4		PCM	34.8	18.8		16.9	22.3	33.8	42.8	50.2
		Other milk	28.7	15.9	ǂ	11.8	17.4	25.5	36.7	50.1		Other milk	34.5	14.6	N.S.	18.8	24.2	32.0	42.8	54.6
		Non-dairy	16.0	11.2	*	4.5	7.7	13.4	22.2	31.3		Non-dairy	24.7	12.7	*	11.0	15.3	22.6	31.2	41.8
Total fat (g)	25–35%E										15–30%E									
		All	19.0	15.9		2.8	7.0	14.9	27.1	40.8		All	21.1	16.7		4.4	9.0	16.6	29.1	44.0
		YCM	25.8	16.4		6.7	13.3	23.6	35.3	45.6		PCM	34.2	19.5		12.9	21.2	29.6	44.8	58.6
		Other milk	25.3	16.5	N.S.	8.1	13.5	20.9	33.1	48.3		Other milk	28.8	16.8	#	11.1	15.9	24.7	39.3	50.9
		Non-dairy	10.3	9.8	*	1.3	3.4	7.5	14.0	22.1		Non-dairy	14.9	12.9	*	2.9	5.9	11.6	19.4	31.5
Carbohydrates (g)	50–69%E										55–79%E									
		All	107.4	58.5		40.3	66.4	97.4	139.8	185.1		All	152.6	66.0		77.9	104.5	143.7	190.8	240.7
		YCM	116.5	54.2		53.6	76.1	110.0	152.8	188.3		PCM	153.2	62.7		80.0	114.6	142.9	189.5	237.7
		Other milk	114.7	54.8	N.S.	56.0	75.6	102.1	141.4	189.4		Other milk	158.6	64.3	N.S.	85.3	110.2	149.3	195.0	248.6
		Non-dairy	96.7	61.9	*	30.9	51.8	84.9	127.4	175.8		Non-dairy	148.9	67.2	N.S.	73.3	100.1	139.2	185.1	236.6
Fibre (g)	6–7										8–10									
		All	2.9	2.3		0.7	1.3	2.3	3.8	5.7		All	4.5	2.7		1.8	2.7	4.0	5.8	8.0
		YCM	2.6	2.0		0.5	1.1	2.1	3.7	5.4		PCM	4.3	2.8		1.6	2.6	3.5	5.5	7.6
		Other milk	2.8	2.0	N.S.	0.8	1.3	2.2	3.7	5.4		Other milk	4.4	2.6	N.S.	1.6	2.6	4.0	5.7	7.8
		Non-dairy	3.1	2.5	#	0.8	1.5	2.4	3.9	6.1		Non-dairy	4.7	2.8	N.S.	1.9	2.7	4.0	5.8	8.2
Sodium (mg)	225 ^2^										300 ^2^									
		All	512.6	499.2		70.2	195.6	405.4	685.1	1036.2		All	686.5	590.8		125.7	290.5	553.0	923.1	1369.2
		YCM	478.1	407.6		85.3	215.6	403.0	621.4	892.3		PCM	756.5	557.5		243.3	410.5	601.5	994.4	1435.9
		Other milk	636.4	491.5	*	175.2	313.0	531.3	834.4	1160.5		Other milk	833.8	622.9	N.S.	256.5	427.6	687.4	1043.2	1527.3
		Non-dairy	430.8	528.8	*	21.5	108.0	294.2	564.5	906.3		Non-dairy	590.9	556.3	*	83.4	215.1	443.2	794.2	1266.9
Calcium (mg)	440										440									
		All	374.6	428.2		36.6	82.5	209.6	518.2	947.5		All	283.1	275.6		73.5	116.7	199.2	342.8	572.3
		YCM	622.3	534.3		57.3	204.7	510.2	890.5	1268.4		PCM	419.8	401.3		67.0	160.6	320.1	546.2	856.9
		Other milk	540.1	425.2	N.S.	134.8	239.3	407.8	697.5	1111.9		Other milk	446.5	310.1	*	187.8	242.4	357.5	524.5	819.2
		Non-dairy	111.5	94.9	*	20.2	44.1	86.6	156.1	220.8		Non-dairy	169.3	148.7	*	61.7	91.6	138.5	203.8	302.2
Phosphorus (mg)	380										405									
		All	395.7	304.2		99.6	183.4	318.9	516.7	788.0		All	460.4	241.2		199.9	289.4	416.2	582.7	768.4
		YCM	407.6	282.6		90.6	206.1	343.1	595.6	800.8		PCM	501.6	275.8		176.0	298.6	487.4	641.1	890.1
		Other milk	574.7	350.0	*	221.5	332.9	488.6	721.8	1074.3		Other milk	593.3	262.0	*	321.9	411.4	536.9	711.1	956.7
		Non-dairy	245.1	163.1	*	69.3	123.0	214.8	328.7	456.8		Non-dairy	377.0	179.6	*	174.3	242.9	346.6	478.2	621.5
Iron (mg)	6.4/7.0										7.5/7.4									
		All	4.4	5.1		0.9	1.6	3.0	5.4	9.7		All	5.2	3.8		1.8	2.7	4.2	6.6	9.1
		YCM	8.7	6.8		2.1	4.2	7.4	11.9	16.5		PCM	8.7	4.8		3.3	5.6	8.3	10.6	15.3
		Other milk	3.4	4.7	*	1.0	1.5	2.6	4.0	6.1		Other milk	5.3	3.9	*	2.0	2.8	4.4	6.8	9.3
		Non-dairy	3.0	2.4	*	0.6	1.3	2.4	4.3	6.1		Non-dairy	4.6	3.2	*	1.6	2.6	3.9	5.8	8.1
Magnesium (mg)	60/60 ^2^										70/70 ^2^									
		All	59.3	48.0		15.7	28.0	47.7	76.9	114.0		All	88.0	60.1		37.6	52.5	76.5	107.9	142.1
		YCM	73.0	50.3		21.0	37.4	62.0	100.2	136.3		PCM	92.6	53.6		34.9	56.5	87.7	121.3	153.3
		Other milk	56.7	53.4	*	16.3	26.0	41.9	68.6	109.2		Other milk	92.0	66.4	N.S.	34.7	52.4	75.8	111.0	154.7
		Non-dairy	54.3	40.2	*	13.6	26.3	45.4	72.2	102.9		Non-dairy	85.0	56.8	N.S.	39.6	52.7	75.9	103.9	132.2
Potassium (mg)	1000 ^2^										1400 ^2^									
		All	481.2	400.3		118.1	218.1	370.4	605.1	994.8		All	627.3	396.4		252.8	357.1	536.5	795.8	1085.5
		YCM	707.7	541.9		156.1	314.5	560.7	1051.0	1351.9		PCM	812.4	525.4		280.2	464.9	718.2	1042.5	1441.4
		Other milk	471.0	355.3	*	164.7	247.1	380.1	567.8	840.6		Other milk	691.6	425.7	#	292.9	406.3	591.6	876.9	1145.8
		Non-dairy	370.8	281.6	*	88.5	164.0	300.9	506.5	769.1		Non-dairy	565.3	342.2	*	239.2	339.8	477.1	704.8	975.8
Selenium (μg)	13.6/13.0										16.1/15.6									
		All	30.7	23.4		7.9	14.2	25.8	40.9	58.9		All	47.1	27.0		17.6	27.1	41.5	61.2	83.5
		YCM	27.8	21.4		5.2	12.1	22.8	39.1	53.0		PCM	48.1	24.3		19.5	30.5	44.9	65.2	79.3
		Other milk	33.7	24.3	*	10.9	16.9	27.6	43.0	62.4		Other milk	50.6	28.6	N.S.	20.0	30.5	44.6	65.1	87.8
		Non-dairy	29.8	23.4	N.S.	7.2	13.3	24.1	39.9	59.0		Non-dairy	44.9	26.1	N.S.	16.5	25.9	39.6	58.7	79.6
Zinc (mg)	2.8/2.6										3.3/3.2									
		All	3.0	2.8		0.7	1.2	2.2	3.7	6.3		All	3.3	2.2		1.2	1.8	2.8	4.3	6.1
		YCM	5.4	3.8		1.3	2.6	4.8	7.4	10.1		PCM	5.6	2.6		2.6	3.8	5.4	6.9	9.0
		Other milk	2.7	1.8	*	1.0	1.5	2.3	3.5	4.8		Other milk	3.7	2.1	*	1.6	2.1	3.2	4.5	6.5
		Non-dairy	1.9	1.8	*	0.4	0.8	1.4	2.5	3.7		Non-dairy	2.8	1.9	*	1.0	1.6	2.4	3.6	4.8
Thiamin (mg)	0.4/0.4										0.5/0.4									
		All	0.4	0.4		0.1	0.2	0.3	0.6	0.9		All	0.5	0.4		0.2	0.3	0.4	0.7	1.0
		YCM	0.7	0.5		0.2	0.3	0.6	0.9	1.2		PCM	0.7	0.5		0.3	0.5	0.7	0.9	1.1
		Other milk	0.4	0.4	*	0.1	0.2	0.3	0.5	0.7		Other milk	0.6	0.4	*	0.2	0.3	0.5	0.7	1.0
		Non-dairy	0.3	0.3	*	0.1	0.1	0.2	0.4	0.7		Non-dairy	0.4	0.3	*	0.1	0.2	0.4	0.6	0.9
Riboflavin (mg)	0.4/0.4										0.5/0.4									
		All	0.7	0.8		0.1	0.2	0.4	1.0	1.6		All	0.6	1.8		0.1	0.2	0.4	0.7	1.1
		YCM	0.9	0.8		0.2	0.3	0.7	1.3	1.9		PCM	0.7	0.5		0.2	0.3	0.6	0.9	1.2
		Other milk	1.1	0.9	*	0.3	0.5	0.9	1.4	2.3		Other milk	1.1	3.1	*	0.4	0.5	0.8	1.1	1.7
		Non-dairy	0.2	0.2	*	0.1	0.1	0.2	0.3	0.5		Non-dairy	0.4	0.3	*	0.1	0.2	0.3	0.5	0.7
Niacin (mg)	5/5										5/5									
		All	5.8	4.1		1.5	2.7	4.9	7.9	11.2		All	8.4	5.1		3.3	5.0	7.4	10.7	14.4
		YCM	7.9	4.5		2.7	4.6	7.2	10.3	14.2		PCM	10.7	5.0		4.8	6.8	10.7	14.0	16.5
		Other milk	5.2	3.8	*	1.8	2.6	4.2	6.5	9.5		Other milk	8.6	6.1	*	3.4	5.2	7.5	10.4	14.4
		Non-dairy	5.1	3.8	*	1.2	2.3	4.2	7.1	10.2		Non-dairy	7.9	4.3	*	3.1	4.8	7.1	10.2	13.7
Vitamin B6 (mg)	0.4/0.5										0.5/0.5									
		All	0.5	0.5		0.1	0.2	0.3	0.6	0.9		All	0.6	0.5		0.2	0.4	0.5	0.8	1.1
		YCM	0.7	0.5		0.2	0.4	0.6	1.0	1.3		PCM	0.9	0.6		0.4	0.5	0.8	1.1	1.5
		Other milk	0.4	0.5	*	0.1	0.2	0.3	0.5	0.7		Other milk	0.7	0.6	*	0.2	0.4	0.5	0.8	1.2
		Non-dairy	0.4	0.3	*	0.1	0.2	0.3	0.5	0.7		Non-dairy	0.6	0.3	*	0.2	0.3	0.5	0.7	1.0
Vitamin B12 (mg)	0.8/0.9										0.9/1.0									
		All	1.2	1.8		0.0	0.2	0.7	1.6	2.7		All	1.9	3.8		0.2	0.5	1.1	2.1	3.8
		YCM	1.5	1.5		0.2	0.6	1.2	2.0	3.0		PCM	1.9	1.7		0.4	0.8	1.5	2.4	3.5
		Other milk	1.2	2.2	*	0.0	0.2	0.7	1.5	2.9		Other milk	1.9	5.0	N.S.	0.2	0.5	1.2	2.1	3.8
		Non-dairy	1.0	1.6	*	0.0	0.1	0.6	1.3	2.4		Non-dairy	1.8	3.2	#	0.1	0.4	1.0	2.1	4.0
Folate (μg)	120/120										160/160									
		All	101.1	106.4		12.6	31.3	69.0	133.4	232.8		All	126.6	104.8		24.6	53.2	99.7	167.8	263.3
		YCM	184.1	153.9		36.7	66.6	140.0	266.1	388.5		PCM	212.7	131.1		75.3	121.2	180.2	292.4	404.0
		Other milk	74.9	66.1	*	11.7	26.5	58.3	98.1	165.4		Other milk	115.9	89.1	*	26.6	52.2	93.1	158.9	234.4
		Non-dairy	78.9	76.5	*	9.6	24.5	55.6	109.7	175.9		Non-dairy	121.5	104.5	*	22.1	48.4	95.0	158.5	255.2
Vitamin C (mg)	12/11										17/17									
		All	21.2	33.3		0.0	2.0	9.2	24.9	58.4		All	19.2	30.4		0.0	2.6	8.6	21.9	47.4
		YCM	50.8	43.9		2.0	18.3	40.1	74.0	109.4		PCM	40.7	43.4		5.5	13.6	28.1	53.2	90.1
		Other milk	14.2	18.2	*	2.0	4.4	9.4	16.8	28.7		Other milk	18.8	25.5	*	2.6	5.0	10.2	20.4	42.4
		Non-dairy	11.4	26.8	*	0.0	0.0	3.4	13.0	29.6		Non-dairy	16.6	29.9	*	0.0	0.6	6.3	18.1	42.0
Vitamin D (μg)	5 ^3^										5 ^3^									
		All	2.1	3.7		0.0	0.1	0.8	2.5	5.9		All	1.7	2.3		0.0	0.3	1.1	2.2	4.1
		YCM	5.9	5.1		0.6	2.3	4.8	8.1	12.4		PCM	4.3	3.6		1.3	2.1	3.3	5.2	8.1
		Other milk	1.0	2.1	*	0.0	0.0	0.5	1.3	2.6		Other milk	1.4	1.5	*	0.0	0.3	1.0	2.0	3.3
		Non-dairy	1.1	2.2	*	0.0	0.0	0.5	1.4	2.6		Non-dairy	1.5	2.3	*	0.0	0.3	0.9	1.9	3.6
Vitamin E (mg)	4 ^3^										4 ^3^									
		All	1.8	2.3		0.1	0.4	1.0	2.2	4.6		All	2.0	2.1		0.4	0.7	1.5	2.6	4.3
		YCM	4.2	3.3		0.5	1.7	3.5	5.8	9.0		PCM	4.7	3.1		1.6	2.5	4.3	6.2	8.9
		Other milk	1.2	1.5	*	0.1	0.3	0.8	1.4	2.5		Other milk	2	1.9	*	0.4	0.8	1.5	2.7	4.1
		Non-dairy	1.1	1.1	*	0.1	0.3	0.8	1.5	2.6		Non-dairy	1.7	1.8	*	0.3	0.6	1.3	2.2	3.3

A represents test for significance between mean intakes of YCM/PCM consumers and Other milk consumers, and YCM/PCM consumers and Non-dairy consumers. N.S. non-significant; # *p* < 0.05; ǂ *p* <0.01; * *p* <0.001. M = Male; F = Female. ^1^ Recommended Energy Intake (REI); ^2^ Recommended Nutrient Intakes (RNI); ^3^ Adequate Intake (AI); Reference intakes w/o superscripts = EAR.

**Table 3:** Percentage of children with inadequate intakes per consumer group by age groups one to two years, three to four years, and all ages.

	1 to 2 Years				3 to 4 Years				All Children			
	% Children Below EAR/AI/AMDR				% Children Below EAR/AI/AMDR				% Children Below EAR/AI/AMDR			
	YCM Consumers	Other milk Consumers	Non-Dairy Consumers	All	PCM Consumers	Other milk Consumers	Non-Dairy Consumers	All	YCM/PCM Consumers	Other milk Consumers	Non-Dairy Consumers	All
Protein	24%	17%	54%	22%	10%	8%	32%	22%	20%	12%	41%	28%
Total fat	36%	40%	83%	37%	4%	13%	56%	37%	28%	27%	67%	47%
Carbohydrates	15%	23%	5%	14%	32%	25%	6%	14%	19%	24%	6%	14%
Calcium	44%	53%	99%	84%	67%	65%	97%	84%	50%	59%	98%	77%
Phosphorus	55%	33%	82%	48%	41%	24%	63%	48%	51%	29%	71%	54%
Iron	44%	92%	92%	81%	43%	80%	87%	81%	44%	86%	89%	81%
Selenium	27%	15%	24%	8%	6%	6%	9%	8%	22%	10%	16%	15%
Zinc	26%	61%	78%	60%	19%	51%	70%	60%	24%	56%	73%	60%
Thiamin	34%	61%	73%	54%	24%	47%	62%	54%	31%	54%	67%	57%
Riboflavin	28%	17%	82%	52%	35%	16%	75%	52%	30%	16%	78%	50%
Niacin	28%	59%	57%	25%	12%	23%	27%	25%	24%	40%	40%	38%
Vitamin B6	34%	73%	68%	48%	21%	49%	51%	48%	31%	61%	58%	55%
Vitamin B12	37%	58%	61%	44%	34%	42%	47%	44%	36%	50%	53%	49%
Folate	49%	82%	83%	73%	45%	76%	75%	73%	48%	79%	78%	74%
Vitamin C	18%	60%	73%	68%	29%	68%	74%	68%	21%	64%	73%	62%
Vitamin D	52%	98%	97%	93%	72%	96%	94%	93%	58%	97%	96%	90%
Vitamin E	56%	96%	97%	88%	64%	95%	97%	94%	58%	96%	97%	91%

**Table 4:** Odds ratios of children meeting the estimated average requirements/adequate intake/Acceptable Macronutrient Distribution Ranges (EAR/AI/AMDR) of each nutrient by consumer group compared to YCM/PCM consumers (reference) in age groups one to two years and three to four years.

	**Other milk Consumers**				**Non-Dairy Consumers**				**Factors Associated with Increased Odds of Meeting EAR/AI/AMDR of Nutrient (*p* < 0.05)**	**Factors Associated with Lower Odds of Meeting EAR/AI/AMDR of Nutrient (*p* < 0.05)**
	**OR**	**95% CI**		***p*** **-Value**	**OR**	**95% CI**		***p*** **-Value**		
Protein	2.27	1.33	3.88	0.003	0.39	0.23	0.63	<0.001	Living in urban dwelling	Education levels: Elementary, high school, No grades completed
Total fat	1.41	0.89	2.24	0.146	0.22	0.13	0.35	<0.001	Wealth quintiles: rich, richest	Education level: Elementary
Carbohydrates	0.35	0.20	0.63	<0.001	1.69	0.83	3.43	0.145	-	Wealth quintiles: richest
Calcium	1.05	0.67	1.66	0.828	0.01	0.01	0.04	<0.001	Wealth quintile: richest	Wealth quintiles: poorest
Phosphorus	3.07	1.94	4.85	<0.001	0.34	0.21	0.54	<0.001	Living in urban dwelling	-
Iron	0.09	0.05	0.16	<0.001	0.08	0.05	0.15	<0.001	-	-
Selenium	2.60	1.49	4.53	0.001	1.70	0.99	2.92	0.054	-	Wealth quintiles: poor, poorest; Education level: No grades completed
Zinc	0.37	0.23	0.59	<0.001	0.17	0.11	0.28	<0.001	Wealth quintile: richest; Living in urban dwelling	Education level: Elementary
Thiamin	0.43	0.27	0.68	<0.001	0.25	0.16	0.41	<0.001	Wealth quintile: richest; Living in urban dwelling	Education level: Elementary
Riboflavin	3.07	1.83	5.17	<0.001	0.14	0.08	0.22	<0.001	-	Education level: Elementary
Niacin	0.35	0.22	0.56	<0.001	0.38	0.24	0.60	<0.001	Living in urban dwelling	Education levels: Elementary, high school
Vitamin B6	0.27	0.17	0.42	<0.001	0.36	0.23	0.56	<0.001	Wealth quintile: richest; Living in urban dwelling	Education levels: Elementary, high school
Vitamin B12	0.51	0.33	0.78	0.002	0.49	0.32	0.77	0.002	-	Education levels: Elementary, high school
Folate	0.22	0.14	0.36	<0.001	0.21	0.13	0.35	<0.001	-	Wealth quintiles: poor, poorest
Vitamin C	0.17	0.10	0.29	<0.001	0.10	0.06	0.16	<0.001	-	-
Vitamin D	0.02	0.01	0.06	<0.001	0.03	0.02	0.08	<0.001	-	-
Vitamin E	0.05	0.02	0.11	<0.001	0.04	0.02	0.09	<0.001	-	Education level: Elementary
	**Other milk Consumers**				**Non-dairy Consumers**				**Factors Associated with Increased Odds of Meeting EAR/AI/AMDR of Nutrient (*p* < 0.05)**	**Factors Associated with Lower Odds of Meeting EAR/AI/AMDR of Nutrient (*p* < 0.05)**
	**OR**	**95% CI**		***p*** **-Value**	**OR**	**95% CI**		***p*** **-Value**		
Protein	1.65	0.57	4.79	0.358	0.51	0.18	1.44	0.204	Living in urban dwelling	-
Total fat	0.53	0.15	1.86	0.320	0.09	0.03	0.32	<0.001	Living in urban dwelling	Wealth quintiles: poor, poorest; Education level: Vocational
Carbohydrates	0.79	0.39	1.60	0.510	2.57	1.19	5.54	0.016	Wealth quintiles: poor, poorest	Wealth quintile: richest; Living in urban dwelling; Mother currently working
Calcium	1.48	0.76	2.88	0.244	0.11	0.05	0.24	<0.001	Living in urban dwelling	-
Phosphorus	2.54	1.32	4.92	0.006	0.65	0.34	1.23	0.186	Wealth quintile: richest; Living in urban dwelling	Education level: Elementary
Iron	0.24	0.12	0.46	<0.001	0.21	0.11	0.41	<0.001	Wealth quintile: richest; Living in urban dwelling	Education level: Vocational
Selenium	2.30	0.55	9.58	0.251	2.24	0.54	9.25	0.266	Living in urban dwelling	Education levels: Elementary, no grades completed
Zinc	0.43	0.20	0.89	0.024	0.26	0.12	0.55	<0.001	Wealth quintiles: rich, richest; Living in urban dwelling	-
Thiamin	0.46	0.22	0.93	0.031	0.36	0.18	0.73	0.005	Living in urban dwelling	Education level: No grades completed
Riboflavin	6.24	3.03	12.84	<0.001	0.55	0.28	1.09	0.087	Wealth quintile: richest; Living in urban dwelling	Education level: Elementary
Niacin	0.95	0.40	2.25	0.912	1.28	0.54	3.06	0.576	Living in urban dwelling	Education level: Elementary
Vitamin B6	0.42	0.21	0.86	0.017	0.44	0.22	0.90	0.024	-	-
Vitamin B12	0.89	0.48	1.67	0.721	0.87	0.46	1.63	0.656	-	Education level: No grades completed
Folate	0.28	0.15	0.53	<0.001	0.30	0.16	0.56	<0.001	-	-
Vitamin C	0.19	0.09	0.37	<0.001	0.14	0.07	0.28	<0.001	Wealth quintile: richest	-
Vitamin D	0.11	0.04	0.27	<0.001	0.14	0.06	0.34	<0.001	-	-
Vitamin E	0.12	0.05	0.27	<0.001	0.09	0.04	0.22	<0.001	-	-

YCM/PCM consumer group was considered as reference group (OR = 1.0) for the respective age groups (one to two years; three to four years).

## Supplementary Materials

The following are available online at https://www.mdpi.com/2072-6643/12/2/392/s1, Table S1: Nutritional compositions of YCMBs, Milk (fluid), Milk (powdered) considered in the current study.
