# Supplementary material for: Contribution of Milk Beverages to Nutrient Adequacy of Young Children and Preschool Children in the Philippines
Source: Nutrients. 2020 Feb 1;12(2):392. doi: 10.3390/nu12020392 (PMC7071197; doi:10.3390/nu12020392)
Supplement: Supplementary file 1 [file nutrients-12-00392-s001.pdf]

Supplementary Materials: The following are available online at www.mdpi.com/xxx/s1, Table S1: Nutritional compositions of YCM/PCM, Milk (fluid), Milk (powdered) considered in the current study

Table S1: Nutritional compositions of YCMBs, Milk (fluid), Milk (powdered) considered in the current study

	YCM/PCM[1]			Milk (Fluid)[2]			Milk (Powdered)[3]		
	Min	Max	Mean	Min	Max	Mean	Min	Max	Mean
Energy (kcal)	410.0	510.0	449.9	43.3	334.2	126.9	321.0	508.0	433.6
Protein (g)	5.8	24.3	14.6	1.2	8.5	4.8	7.9	40.8	24.3
Total fat (g)	5.3	28.8	16.5	0.2	9.5	4.3	0.8	28.0	17.2
Carbohydrates (g)	38.0	72.0	59.0	4.3	59.3	17.4	37.8	54.4	45.5
Fibre (g)	0.0	3.2	1.6	0.0	0.8	0.1	0.0	3.0	0.6
Sodium (mg)	0.0	300.0	185.7	21.7	127.0	71.7	127.0	495.8	332.7
Calcium (mg)	483.0	940.0	734.4	39.0	371.0	188.0	284.0	1318.0	877.5
Phosphorus (mg)	0.0	740.0	392.5	30.0	253.0	124.7	253.0	949.0	661.1
Iron (mg)	5.6	10.0	7.4	0.1	0.8	0.3	0.2	5.4	1.8
Magnesium (mg)	0.0	104.0	54.1	0.0	96.4	25.8	0.0	110.1	66.4
Potassium (mg)	0.0	1400.0	632.3	2.1	497.1	233.0	0.0	1641.9	803.5
Selenium (µg)	0.0	15.0	6.2	0.0	19.8	4.4	0.0	16.2	6.6
Zinc (mg)	0.0	7.0	4.3	0.1	1.6	0.6	0.0	4.4	2.4
Thiamin (mg)	0.0	1.4	0.5	0.0	0.7	0.1	0.3	1.7	0.8
Riboflavin (mg)	0.1	1.8	0.9	0.1	0.7	0.3	0.8	1.7	1.4
Niacin (mg)	0.0	12.4	5.2	0.0	3.6	0.4	0.3	32.7	7.1
Vitamin B6 (mg)	0.0	1.3	0.5	0.0	1.4	0.2	0.0	0.4	0.1
Vitamin B12 (mg)	0.0	2.8	1.2	0.0	2.9	0.5	0.0	3.8	1.6
Folate (µg)	0.0	383.3	161.4	0.0	18.0	6.5	0.0	166.7	40.7
Vitamin C (mg)	0.0	81.0	46.8	0.0	4.0	1.0	0.0	104.0	27.6
Vitamin D (µg)	3.5	9.6	5.7	0.0	1.0	0.2	0.0	2.4	0.6
Vitamin E (mg)	0.0	7.7	3.9	0.0	0.7	0.1	0.0	4.0	1.1

[1] YCM/PCM includes 18 brands; [2] Milk (Fluid) includes evaporated milk, condensed milk, buttermilk, as well as cow's and goat's milk; [3] Milk (Powdered) includes skimmed, full cream, filled milk, and branded full fat powdered milks
